# Geochemical and Microbiological Evidence for Microbial Methane Production in Deep Aquifers of the Cretaceous Accretionary Prism

**DOI:** 10.1264/jsme2.ME17199

**Published:** 2018-06-13

**Authors:** Makoto Matsushita, Kenta Magara, Yu Sato, Naoya Shinzato, Hiroyuki Kimura

**Affiliations:** 1 Department of Environment and Energy Systems, Graduate School of Science and Technology, Shizuoka University 836 Oya, Suruga-ku, Shizuoka 422–8529 Japan; 2 Department of Science, Graduate School of Integrated Science and Technology, Shizuoka University 836 Oya, Suruga-ku, Shizuoka 422–8529 Japan; 3 Tropical Biosphere Research Center, Ryukyus University 1 Senbaru, Nishihara, Okinawa 903–0213 Japan; 4 Department of Geosciences, Faculty of Science, Shizuoka University 836 Oya, Suruga-ku, Shizuoka 422–8529 Japan; 5 Research Institute of Green Science and Technology, Shizuoka University 836 Oya, Suruga-ku, Shizuoka 422–8529 Japan

**Keywords:** accretionary prism, deep aquifer, methanogenic archaea, fermentative bacteria, syntrophic consortium

## Abstract

Accretionary prisms are thick layers of sedimentary material piled up at convergent plate boundaries. Large amounts of anaerobic groundwater and methane (CH_4_) are contained in the deep aquifers associated with accretionary prisms. In order to identify microbial activity and CH_4_ production processes in the deep aquifers associated with the Cretaceous accretionary prism in Okinawa Island, Japan, we performed geochemical and microbiological studies using anaerobic groundwater and natural gas (mainly CH_4_) samples collected through four deep wells. Chemical and stable hydrogen and oxygen isotope analyses of groundwater samples indicated that the groundwater samples obtained from each site originated from ancient seawater and a mixture of rainwater and seawater, respectively. Additionally, the chemical and stable carbon isotopic signatures of groundwater and natural gas samples suggested that CH_4_ in the natural gas samples was of a biogenic origin or a mixture of biogenic and thermogenic origins. Microscopic observations and a 16S rRNA gene analysis targeting microbial communities in groundwater samples revealed the predominance of dihydrogen (H_2_)-producing fermentative bacteria and H_2_-utilizing methanogenic archaea. Moreover, anaerobic cultures using groundwater samples suggested a high potential for CH_4_ production by a syntrophic consortium of H_2_-producing fermentative bacteria and H_2_-utilizing methanogenic archaea through the biodegradation of organic substrates. Collectively, our geochemical and microbiological data support the conclusion that the ongoing biodegradation of organic matter widely contributes to CH_4_ production in the deep aquifers associated with the Cretaceous accretionary prism.

Accretionary prisms are thick layers of sedimentary material piled up at convergent plate boundaries. These materials originated from ancient marine sediments that were deposited on a subducting ocean plate and accreted onto a non-subducting continental plate (47). Accretionary prisms are found in large regions of the world, including Alaska and Washington in the U.S., New Zealand, Chile, Peru, Indonesia, Taiwan, Russia, and Japan (15, 19, 23, 26).

The Shimanto Belt in southwest Japan is a typical and highly studied accretionary prism. The Shimanto Belt was mainly formed during the Cretaceous and Paleogene Periods and originated from ancient marine sediments that were deposited on the Philippine Sea Plate (23). These sediments are approximately 10 km thick and traceable laterally for 1,800 km in parallel with the Nankai Trough and Ryukyu Trench (Fig. 1A) (46). They are mainly composed of non- to weakly metamorphosed sequences of sandstone, mudstone, chert, and greenstone. Groundwater is primarily recharged by rainwater and seawater that infiltrates into outcrops or faults, then flows down through permeable sandstone and is anaerobically reserved in deep aquifers. In addition to anaerobic groundwater, a high concentration of natural gas, mainly methane (CH_4_), is contained in deep aquifers (27, 32, 43).

It is generally accepted that the origin of CH_4_ in natural gas reserves in subsurface sedimentary deposits is either biogenic (formed by methanogenic archaea) or thermogenic (formed by the thermal degradation of organic matter in sedimentary layers). Previous studies performed a series of geochemical and microbiological analyses of anaerobic groundwater and natural gas derived from deep aquifers of the Paleogene accretionary prism distributed in southwest Japan. Based on their findings, these studies suggested a syntrophic consortium model in deep aquifers, in which the anaerobic biodegradation of organic matter in the sediment mediated by H_2_-producing fermentative bacteria and H_2_-utilizing methanogenic archaea contribute to CH_4_ production (4, 27, 32).

The anaerobic deep aquifers associated with accretionary prisms are considered to contain large amounts of CH_4_. This CH_4_ is a potential greenhouse gas and important energy resource. However, all of the research on the CH_4_ production process conducted to date has targeted the Paleogene accretionary prism. Although Cretaceous accretionary prisms are also distributed across large regions of the world, microbial activity and CH_4_ production processes in the deep aquifers of older accretionary prisms remain unknown.

Therefore, the objectives of the present study were to reveal the microbial activity and CH_4_ production process in deep aquifers associated with the Cretaceous accretionary prism in Okinawa Island, Japan. We collected anaerobic groundwater and natural gas samples derived from deep aquifers through deep wells, and subjected them to a series of geochemical and microbiological analyses. The microbial activity and CH_4_ production process revealed in the present study were compared with those previously reported in deep aquifers of the Paleogene accretionary prism.

## Materials and Methods

### Study site and sample collection

**Fig. 1:**
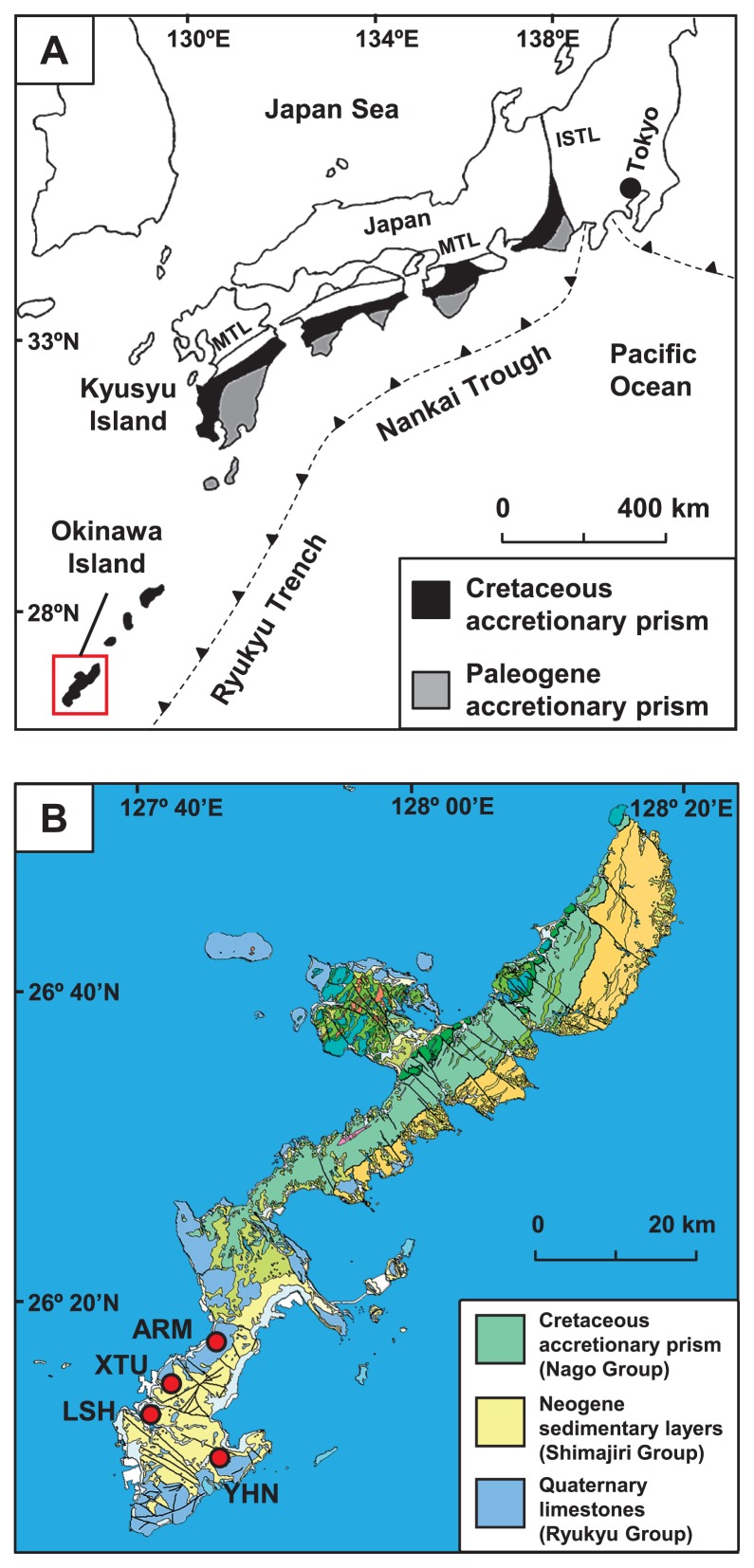
(A) The location of the accretionary prism in Japan, known as the Shimanto Belt, and (B) geological map of the study area (red square). The location of the Shimanto Belt was taken from Kano *et al.* (23). The geological map is modified from a 1:200,000 seamless digital geological map of Japan (16). The red circles indicate the location of the wells used for sampling. MTL, Median Tectonic Line; ISTL, Itoigawa-Shizuoka Tectonic Line.

Okinawa Island has an area of 1,207 km^2^ and is located approximately 640 km south of Kyusyu Island, Japan (Fig. 1A). The accretionary prism known as the Nago Group (mainly Cretaceous) in the Shimanto Belt is distributed over the island (Fig. 1B) (35, 50). The Nago Group is composed principally of sandstone, mudstone, and greenstone. In the southern part of Okinawa Island, the sedimentary layers referred to as the Shimajiri Group (mainly Neogene) unconformably overlay the Nago Group (49). The Shimajiri Group is composed mainly of sandstone, mudstone, and tuff, and is unconformably covered with Quaternary limestone (the Ryukyu Group).

Anaerobic groundwater and natural gas samples derived from deep aquifers in the Nago Group were collected through four deep wells: YHN, LSH, XTU, and ARM (Fig. 1B). These wells were drilled down to 800–2,119 m and reached deep aquifers in the Nago Group (Table S1) (25, 49). As well strainers extend over the Nago and Shimajiri Groups at the YHN site, samples may also contain groundwater and natural gas derived from the Shimajiri Group.

Groundwater at these wells is anaerobically drawn up to ground level by a water pump or by natural water pressure. In the present study, groundwater was pumped for more than 24 h before sampling in order to prevent contamination by air and water from shallow environments. Groundwater samples were collected under anaerobic conditions into autoclaved serum bottles and polycarbonate bottles using a sterile silicone tube. The concentrations of dissolved natural gas were so high that gas exsolved at the ground level. Natural gas samples were collected into autoclaved serum bottles underwater in order to prevent contamination by air. Serum bottles were tightly sealed with sterile butyl-rubber stoppers and aluminum crimps.

### Physicochemical and stable isotope analyses of groundwater and natural gas

We measured the temperature, pH, oxidation-reduction potential (ORP), and electrical conductivity (EC) of groundwater samples at the outflow of the wells. The concentrations of cations (Na^+^, Ca^2+^, Mg^2+^, K^+^, and NH_4_^+^) and anions (CI^−^, Br^−^, I^−^, F^−^, PO_4_^3−^, NO_3_^−^, SO_4_^2−^, HCO_3_^−^, acetate, and formate) in groundwater were analyzed using an ICS-1500 ion chromatography system (Dionex, Sunnyvale, CA, USA). Sulfide was analyzed using a No. 211 sulphide ion detector tube (Gastec, Kanagawa, Japan). Dissolved organic carbon (DOC) was measured on a TOC-V total organic carbon analyzer (Shimadzu, Kyoto, Japan). The concentrations of natural gas components (H_2_, N_2_, O_2_, CO_2_, CH_4,_ C_2_H_6_, C_3_H_8_, and C_4_H_10_) were analyzed on a GC-2014 gas chromatograph equipped with a thermal conductivity detector and flame ionization detector (Shimadzu) following the procedures described by Matsushita *et al.* (32). The detection limits of the analysis were 0.01 vol.% for H_2_, N_2_, O_2_, CO_2_, and CH_4_, and 0.001 vol.% for C_2_H_6_, C_3_H_8_, and C_4_H_10_.

The stable hydrogen and oxygen isotope ratios of groundwater samples (D/H and ^18^O/^16^O) were measured on a DLT-100 liquid water isotope analyzer (Los Gatos Research, San Jose, CA, USA) (13). The stable carbon isotope ratio (^13^C/^12^C) of dissolved inorganic carbon (DIC; consisting mainly of HCO_3_^−^) was analyzed as described previously (36). Groundwater samples for analyzing the ^13^C/^12^C of DIC were fixed with 0.5 mL of saturated HgCl_2_ solution and sealed with sterile butyl-rubber stoppers and aluminum crimps with no air bubbles. A 10-mL headspace was created inside each serum bottle with pure helium gas and acidified by adding CO_2_-free H_3_PO_4_ solution. Sample bottles were left in the dark for 24 h in order to achieve equilibrium between dissolved CO_2_ and headspace CO_2_. CO_2_ in this headspace was subsampled, and the ^13^C/^12^C ratio of CO_2_ was measured with a Trace GC Ultra gas chromatograph (Thermo Fisher Scientific) that was connected to a Delta^plus^ XL isotope ratio mass spectrometer (IRMS) (Thermo Fisher Scientific). The ^13^C/^12^C of CH_4_ in natural gas was measured with a Flash EA1112 elemental analyzer (Thermo Fisher Scientific) that was connected to a Delta V Advantage IRMS with a Conflo IV interface (Thermo Fisher Scientific). Stable isotope ratios were expressed in the conventional δ notation calculated from the equation:

$$\delta =[R_{\text{sample}}/R_{\text{standard}}-1]\times 1000\;[‰],$$

where *R* is the isotope ratio (D/H, ^18^O/^16^O, or ^13^C/^12^C). All isotope ratios are reported relative to international standards: Vienna Standard Mean Ocean Water for δD and δ^18^O, and Vienna Pee Dee Belemnite for δ^13^C. The standard deviations of δD and δ^18^O in groundwater and δ^13^C of DIC and CH_4_ were ±0.5‰, ±0.1‰, ±1‰, and ±0.3‰, respectively.

### Total cell count and catalyzed reporter deposition fluorescence *in situ* hybridization (CARD-FISH)

Groundwater samples for the total cell count and CARD-FISH analysis were filtered using polycarbonate membrane filters (pore size, 0.2 μm; diameter, 25 mm) (Millipore, Billerica, MA, USA). In the total cell count, microbial cells trapped on the filters were stained with SYBR Green I (Life Technologies, Carlsbad, CA, USA) (55). Stained cells were observed under a BX51 epifluorescence microscope equipped with a U-MNIB3 fluorescence filter (Olympus, Tokyo, Japan), and more than 20 microscopic fields were counted for each sample. Cell counting was performed within 48 h of groundwater sampling.

A CARD-FISH analysis targeting prokaryotic 16S rRNAs was conducted with minor modifications to the protocol described by Matsushita *et al.* (32). Briefly, microbial cells collected on the filter were fixed in 3% paraformaldehyde at 4°C for 1 h and dehydrated in 50, 80, and 99.5% ethanol solutions for 3 min each time. Cell fixation was conducted within 12 h of groundwater sampling. The filter was incubated in a lysozyme solution (5 mg mL^−1^ in 1 mM EDTA, 10 mM Tris-HCl, and 10 mM NaCl) at 37°C for 1 h for cell wall permeabilization. Hybridization was conducted using the following horseradish peroxidase-labeled probes: *Archaea*-specific ARCH915 (45), *Bacteria*-specific EUB338 (3), *Methanobacteriales-*specific MB1174 (39), *Methanomicrobiales*-specific MG1200 (39), *Methanosarcinales*-specific MSMX860 (39), and the control probe Non338 (51) with hybridization buffer described by Mitsunobu *et al.* (34) (35% formamide concentration). The Cy3-labeled tyramide signal was amplified using the TSA-Plus cyanine 3 system (Perkin Elmer, Waltham, MA, USA). All microbial cells were counterstained with SYBR Green I (Life Technologies). Stained cells were observed under a model BX51 epifluorescence microscope (Olympus) equipped with a U-MNIB3 filter (Olympus) for SYBR Green I-stained cells and a U-MWIG3 filter (Olympus) for FISH-positive cells, and more than 20 microscopic fields were counted for each sample.

### Next generation sequencer (NGS) analysis of archaeal and bacterial 16S rRNA genes

In order to analyze archaeal and bacterial populations in groundwater samples, 10 L of each groundwater sample was aseptically filtered using Sterivex-GV filter units (pore size, 0.22 μm) (Millipore). Bulk DNA was extracted from microbial cells trapped on the filter using the MORA-Extract kit (Kyokuto Pharmaceutical, Tokyo, Japan). The V3–V4 region of archaeal and bacterial 16S rRNA genes was simultaneously amplified from bulk DNA by PCR using the primer set, Pro341F and Pro806R (48). Library generation and sequencing using an Illumina MiSeq sequencer were performed according to the method described by Takahashi *et al.* (48). The Ribosomal Database Project Classifier version 2.10 was used to analyze sequence reads (confidence threshold of 80%) (52). Sequence reads were grouped into operational taxonomic units (OTUs) sharing more than 97% sequence similarity, and then the coverage, Chao 1, and Shannon index were calculated using the Quantitative Insights Into Microbial Ecology version 1.5.0 pipeline (10).

### Anaerobic culture of microbial communities in groundwater

Thirty milliliters of each groundwater sample was anaerobically injected into an autoclaved 70-mL serum bottle that was tightly sealed with a sterile butyl-rubber stopper and aluminum crimp. In order to assess the potential for CH_4_ production by methanogenic archaea, groundwater samples were amended with acetate (20 mM), methanol (20 mM), formate (20 mM), or H_2_/CO_2_ (80:20, v/v; 150 kPa). Except in the case of H_2_/CO_2_ amended bottles, the headspaces of the serum bottles were filled with pure N_2_ at 150 kPa. These cultures were anaerobically incubated without shaking at the temperatures of the groundwater samples measured at the outflow of the wells.

In order to measure the potential for H_2_ and CO_2_ production by H_2_-producing fermentative bacteria, groundwater samples were amended with 3 mL of YPG medium (10 g yeast extract, 10 g peptone, and 2 g glucose L^−1^ distilled water) and 2-bromoethanesulfonate (BES, 20 mM), a methanogenesis inhibitor (18). The headspaces of the serum bottles were filled with pure N_2_ at 150 kPa, and these cultures were anaerobically incubated without shaking at the temperatures of the groundwater samples measured at the outflow of the wells.

In order to assess the potential for CH_4_ production by a syntrophic consortium of H_2_-producing fermentative bacteria and H_2_-utilizing methanogenic archaea, groundwater samples were amended with 3 mL of YPG medium. As a killed control, groundwater samples were autoclaved and then amended with 3 mL of YPG medium. The headspaces of the serum bottles were filled with pure N_2_ at 150 kPa, and these cultures were anaerobically incubated without shaking at the temperatures of the groundwater samples measured at the outflow of the wells.

All cultures were performed in duplicate. H_2_, N_2_, CH_4_, and CO_2_ concentrations in the headspaces were measured on a GC-2014 gas chromatograph equipped with a thermal conductivity detector (Shimadzu) as described above.

Archaeal and bacterial 16S rRNA genes in the cultures in which CH_4_ production was observed were analyzed according to the clone library method. Briefly, cells in the cultures were collected by centrifugation and lysed by lysozyme and proteinase K. Bulk DNA was purified using both phenol/chloroform/isoamyl alcohol and chloroform/ isoamyl alcohol and concentrated with ethanol precipitation. Archaeal and bacterial 16S rRNA gene fragments were amplified by PCR from bulk DNA using the *Archaea*-specific primer set, 109aF and 915aR (17, 45), and *Bacteria*-specific primer set, 8bF and 1512uR (14). The sequences of the inserted PCR products selected from recombinant colonies were elucidated with an Applied Biosystems 3730xl DNA analyzer (Life Technologies). A 3% distance level between sequences was considered the cut-off for distinguishing distinct OTUs. The nearest relative of each OTU was identified using the BLAST program (2), and neighbor-joining phylogenetic trees were then constructed using the CLUSTAL X version 2.1 program (29).

### Nucleotide sequence accession numbers

The 16S rRNA gene sequences obtained in the present study have been deposited under DDBJ/ENA/GenBank accession numbers LC179566 to LC179584 and DRA005250.

## Results

### Physicochemical parameters of groundwater and natural gas

The temperature and pH of groundwater samples measured at the outflow of the wells ranged between 40.7 and 53.7°C and between 7.1 and 7.8, respectively (Table 1). The ORP of groundwater ranged between −275 and −179 mV, suggesting anoxic conditions in deep aquifers. The EC, an indicator of salinity, ranged between 878 and 4,500 mS m^−1^. The highest concentrations of Na^+^, Ca^2+^, Mg^2+^, NH_4_^+^, Cl^−^, Br^−^, I^−^, SO_4_^2−^, and DOC were detected in the groundwater sample from the YHN site (Table S2). The concentrations of DOC and HCO_3_^−^ ranged between <0.3 and 17 mg L^−1^ and between 100 and 450 mg L^−1^, respectively. PO_4_^3−^, NO_3_^−^, S^2−^, acetate, and formate were below the limits of detection.

The natural gas samples collected from all sites consisted mainly of CH_4_ (Table 1). The other principal components of natural gas samples were N_2_ and C_2_H_6_. C_3_H_8_ was only detected in the natural gas samples collected from YHN. The hydrocarbon gas composition C_1_/(C_2_+C_3_) of the natural gas samples (C_1_, CH_4_; C_2_, C_2_H_6_; C_3_, C_3_H_8_) ranged between 2,117 and 7,902. The concentrations of H_2_, O_2_, CO_2_, and C_4_H_10_ were below the limits of detection.

### Stable isotopic signatures of groundwater and natural gas

**Fig. 2:**
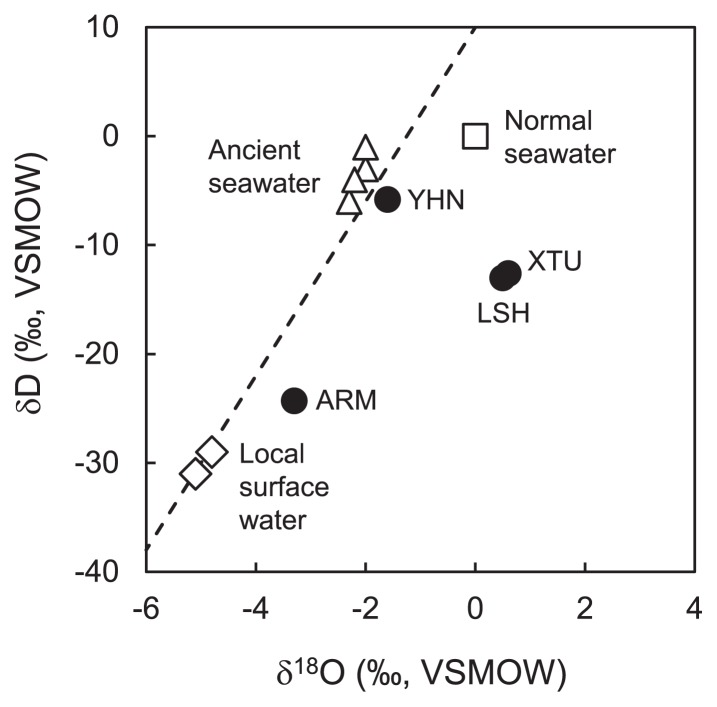
Stable hydrogen and oxygen isotopic composition of groundwater samples along with those of normal seawater (□), ancient seawater (△), and local surface water (⋄). The δD and δ^18^O values of local surface water and ancient seawater were as reported by Agate *et al.* (1) and Maekawa *et al.* (31), respectively. The broken line represents the global meteoric water line (11).

The δD and δ^18^O of groundwater samples ranged between −24.3‰ and −5.8‰ and between −3.3‰ and 0.6‰, respectively (Table S3). In order to estimate the origin of groundwater in deep aquifers, we plotted these δD and δ^18^O values in a δD versus δ^18^O diagram with those of normal seawater, ancient seawater (31), and local surface water (1) and the global meteoric water line (11) (Fig. 2). In this diagram, groundwater collected from YHN was plotted closer to ancient seawater. On the other hand, groundwater from ARM was plotted closer to local surface water. Groundwater from XTU and LSH had similar isotopic signatures and fell on the right region of the global meteoric water line, showing a large δ^18^O enrichment.

**Fig. 3:**
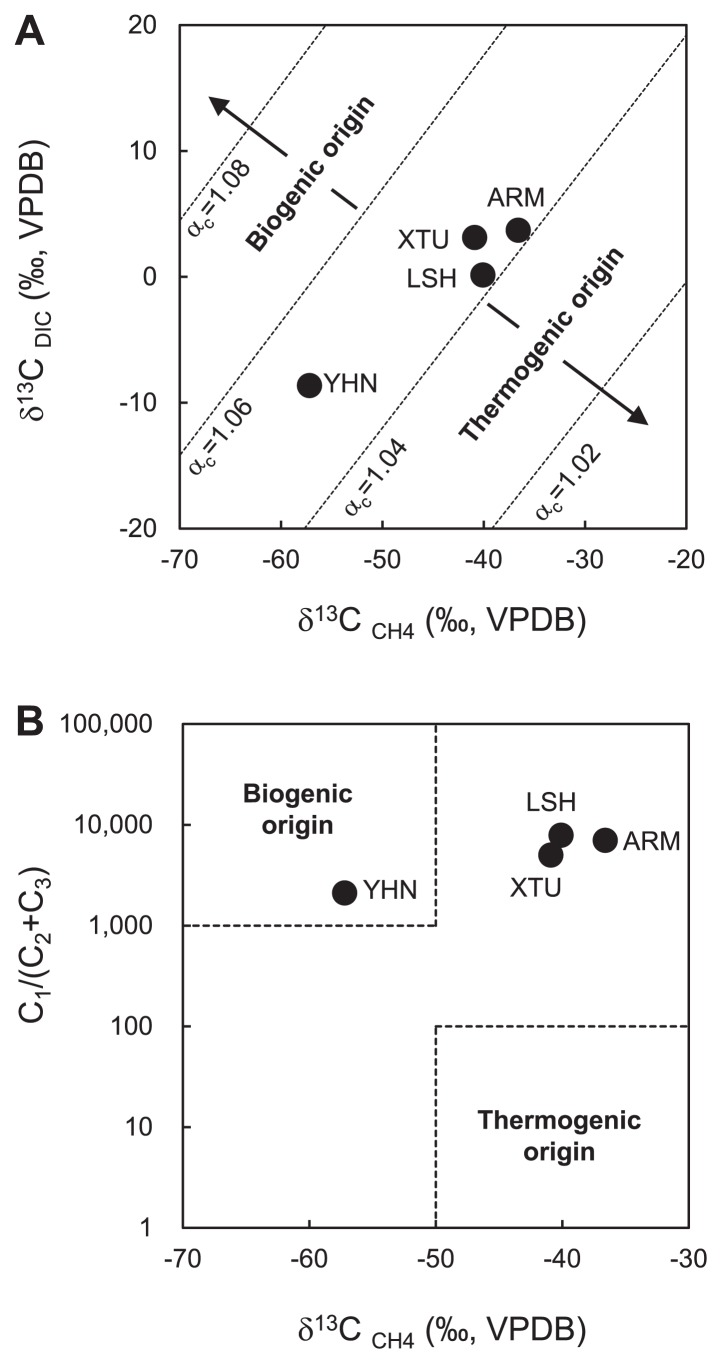
(A) Stable carbon isotope composition of CH_4_ in natural gas samples and dissolved inorganic carbon (DIC) in groundwater samples. The categorization of CH_4_ origins was made according to Smith and Pallasser (45). Dashed lines: equal carbon isotopic fractionation, α_c_=(δ^13^C_DIC_+10^3^)/(δ^13^C_CH4_+10^3^), for α_c_=1.02, 1.04, 1.06, and 1.08. (B) Stable carbon isotope composition of CH_4_ and hydrocarbon gas composition C_1_/(C_2_+C_3_) in natural gas samples. CH_4_ origins were categorized according to Bernard *et al.* (7). VPDB, Vienna Pee Dee Belemnite.

The δ^13^C of DIC in groundwater (δ^13^C_DIC_) ranged between −8.64‰ and 3.70‰ (Table S3). The δ^13^C of CH_4_ in natural gas (δ^13^C_CH4_) ranged between −57.2‰ and −36.6‰. The carbon isotope fractionation (α_c_) between δ^13^C_DIC_ and δ^13^C_CH4_ was 1.042–1.052. In order to estimate the origin of CH_4_ in natural gas samples, we plotted stable isotopic values on the δ^13^C_DIC_ versus δ^13^C_CH4_ diagram described by Smith and Pallasser (44). In this diagram, all samples fell within the boundary between a biogenic origin (α_c_=1.06–1.08) and thermogenic origin (α_c_=1.02–1.04), suggesting that all natural gas samples contained CH_4_ of a mixture of biogenic and thermogenic origins (Fig. 3A). We also used a δ^13^C_CH4_ versus hydrocarbon gas composition C_1_/(C_2_+C_3_) diagram according to Bernard *et al.* (7) in order to estimate the origin of CH_4_ in natural gas samples. In this diagram, the sample from YHN fell within the region of a biogenic origin (Fig. 3B). On the other hand, all other samples fell within the boundary between biogenic and thermogenic origins.

### Abundance of microbial cells in groundwater

Microbial cell densities in anaerobic groundwater samples ranged between 3.4×10^3^ and 1.2×10^5^ cells mL^−1^ (Table 2). In order to detect archaeal and bacterial cells in groundwater samples, we conducted a CARD-FISH analysis targeting archaeal and bacterial 16S rRNAs. FISH-positive archaeal and bacterial cells were detected in groundwater samples from LSH, XTU, and ARM (Fig. S1 and S2), and ranged between 3.7% and 17.4% and between 4.8% and 33.1% of all microbial cells, respectively (Table 2). The ratio of FISH-positive bacterial cells to archaeal cells (*Bacteria*/*Archaea*) was 1.2–2.5. The detection of FISH-positive cells in groundwater obtained from YHN was not possible due to the high autofluorescence of mineral particles in the sample.

In order to detect the cells of methanogenic archaea in groundwater samples, we also performed a CARD-FISH analysis targeting 16S rRNAs specific for the archaeal members of the orders *Methanobacteriales*, *Methanomicrobiales*, and *Methanosarcinales*. FISH-positive *Methanobacteriales* cells were detected in the groundwater obtained from ARM (Fig. S3), and constituted 5.9% of all microbial cells (Table 2). On the other hand, FISH-positive *Methanobacteriales* cells were not confirmed in groundwater from LSH and XTU despite several attempts. FISH-positive *Methanomicrobiales* and *Methanosarcinales* cells were not detected in any of the groundwater samples.

### Microbial community structures in groundwater

In order to identify microbial community structures in groundwater samples, we performed a NGS analysis targeting archaeal and bacterial 16S rRNA genes. We obtained 14,005–51,920 reads and 196–504 OTUs (Table S4). Coverage reached >99.4%. The Chao1 and Shannon index were 231–742 and 2.97–5.62, respectively.

**Fig. 4:**
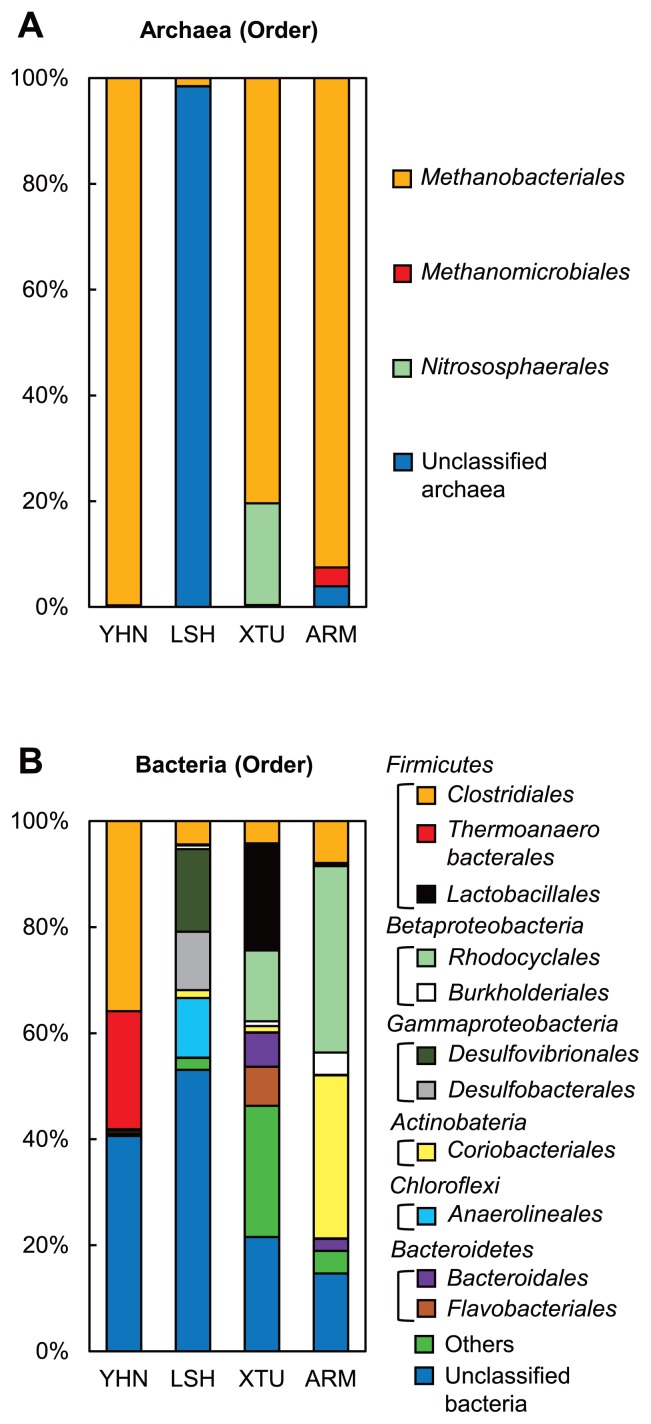
Archaeal and bacterial assemblages in groundwater samples. (A) The relative abundance (%) of archaeal communities. (B) The relative abundance (%) of bacterial communities.

Archaeal 16S rRNA genes accounted for 1.5%–74.0% of the total reads obtained from each sample (Fig. S4). A phylogenetic analysis of the archaeal 16S rRNA genes revealed the predominance of H_2_-utilizing methanogens belonging to the order *Methanobacteriales* in groundwater samples from YHN, XTU, and ARM (Fig. 4A) (56). In LSH, the presence of *Methanobacteriales* was also confirmed. However, most of the archaeal 16S rRNA genes were unclassified archaea. H_2_-utilizing methanogens belonging to the order *Methanomicrobiales* were only identified in ARM (42).

An analysis of bacterial 16S rRNA genes demonstrated the presence of bacterial groups that belong to the phyla *Firmicutes*, *Proteobacteria*, *Actinobacteria*, *Chloroflexi*, and *Bacteroidetes* in groundwater samples from each site (Fig. 4B). Bacterial 16S rRNA genes closely related to *Clostridiales*, a bacterial order that belongs to the *Firmicutes*, were detected in all sites. The orders *Thermoanaerobacterales* and *Lactobacillales*, which are the other members of *Firmicutes*, were mainly identified in YHN and XTU, respectively. The bacterial 16S rRNA genes closely related to the bacterial groups of *Gammaproteobacteria* and *Chloroflexi* were detected in LSH. The bacterial groups of *Betaproteobacteria* and *Actinobacteria* were mainly identified in ARM. The presence of *Bacteroidetes* was shown in XTU and ARM.

### Potential for biogas production by microbial communities

In order to assess the potential for CH_4_ production by methanogenic archaea in the deep aquifers, we anaerobically incubated groundwater samples amended with methanogenic substrates: acetate, methanol, formate, or H_2_/CO_2_. However, CH_4_ production was not observed in these cultures over 75 d of incubation (data not shown).

**Fig. 5:**
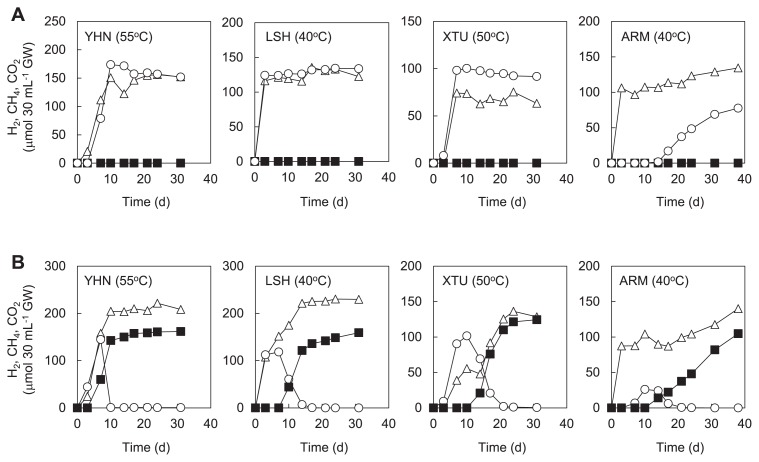
Biogas production from groundwater samples amended with (A) YPG medium and BES and (B) YPG medium incubated at temperatures of groundwater samples measured at the outflow of the well. Cumulative measurements in the gas phase of bottled cultures are shown as follows: H_2_ (○); CH_4_ (■); and CO_2_ (△). Incubation temperatures are shown in parentheses. Although representative results of cultures performed in duplicate are shown, the other culture showed a similar potential for biogas production.

We then performed anaerobic cultivations using groundwater samples amended with YPG medium and BES to assess the potential for H_2_ and CO_2_ production mediated by H_2_-producing fermentative bacteria. As a result, H_2_ and CO_2_ were detected in the gas phase of cultures using groundwater samples from all sites (Fig. 5A). In the cultures using groundwater from LSH and XTU, the production of H_2_ and CO_2_ was observed within 3 d. In the cultures using groundwater from YHN and ARM, H_2_ and CO_2_ were detected after 7 and 14 d, respectively.

A high potential for CH_4_ production was confirmed in the cultures using groundwater samples amended with YPG medium (Fig. 5B). H_2_ and CO_2_ production was observed in all cultures within 7 d. After H_2_ and CO_2_ production, the concentration of H_2_ decreased to below the limit of detection. CH_4_ production was observed after H_2_ levels began to fall. These dynamics of H_2_ and CH_4_ were similar to those observed previously in syntrophic co-cultures of H_2_-producing fermentative bacteria and H_2_-utilizing methanogenic archaea (4, 27, 32). In the cultures using groundwater from YHN and LSH, CH_4_ production was observed within 10 d. In the cultures using groundwater from XTU and ARM, CH_4_ was detected after 14 d. In a killed control using autoclaved groundwater samples amended with YPG medium, H_2_ and CH_4_ production was not observed over 75 d (data not shown).

In order to identify prokaryotes that generated biogas (*i.e.*, H_2_, CO_2_, and CH_4_) in the cultures using YPG-amended groundwater, we constructed archaeal and bacterial 16S rRNA gene clone libraries. The 16S rRNA gene analysis suggested that H_2_-utilizing methanogenic archaea and H_2_-producing fermentative bacteria were predominant in the microbial population (Table S5) and were related to the archaeal order *Methanobacteriales* (Fig. S5) and bacterial orders *Bacillales*, *Synergistales*, *Clostridiales*, and *Thermotogales* (Fig. S6) (12, 30, 40, 53).

## Discussion

The anaerobic groundwater sample collected from the YHN site had a similar EC value and Na^+^ and Cl^−^ concentrations as normal seawater (Tables 1 and S2). The groundwater sample also had high levels of I^−^ and Br^−^. These chemical features are consistent with those of ancient seawater, which is comprised of groundwater that originated from seawater and was maintained for a long period in a low-temperature deep aquifer (24). Additionally, the δD and δ^18^O values of the groundwater sample were consistent with those of ancient seawater (Fig. 2). Therefore, the groundwater in the YHN deep aquifer appeared to have originated from seawater that was conserved in the deep aquifer over a long period at a relatively low temperature. In contrast, groundwater from the ARM site had the lowest EC value (Table 1) and showed similar δD and δ^18^O signatures to those of local surface water (Fig. 2). These characteristics suggest that the ARM deep aquifer has mainly been affected by rainwater infiltrating from surface environments. Groundwater from the LSH and XTU sites had similar chemical and isotopic signatures. The EC value of groundwater was approximately 60% of that of normal seawater (approximately 5,000 mS m^−1^), suggesting that groundwater originated from a mixture of seawater and rainwater (Table 1). The δ^18^O values of the groundwater samples were higher than that of normal seawater sample (Fig. 2). These high δ^18^O values suggest that groundwater in these deep aquifers was affected by water-rock interactions in high-temperature deep subterranean environments (9).

CH_4_ was the predominant component of natural gas samples collected from all sites (Table 1). In the present study, we estimated the origin of CH_4_ in natural gas samples using a δ^13^C_DIC_ versus δ^13^C_CH4_ diagram and δ^13^C_CH4_ versus C_1_/(C_2_+C_3_) diagram (Fig. 3). These chemical and stable carbon isotopic signatures of groundwater and natural gas samples suggested that CH_4_ in the natural gas samples was of a biogenic origin or a mixture of biogenic and thermogenic origins.

The microbial cell densities in groundwater samples were consistent with those previously reported in deep aquifers associated with the Paleogene accretionary prism (27, 32). The CARD-FISH analysis targeting archaeal 16S rRNA detected metabolically active archaeal cells in groundwater samples from LSH, XTU, and ARM (Table 2). Additionally, we successfully detected FISH-positive *Methanobacteriales* cells, known as H_2_-utilizing methanogenic archaea, in the groundwater from ARM. The NGS analysis of archaeal 16S rRNA genes revealed the presence of *Methanobacteriales* in the groundwater from all sites (Fig. 4A). In contrast, methanogenic archaea that use acetate or methanol as methanogenic substrates were not confirmed. These results suggest that H_2_-utilizing methanogenesis is a main microbial CH_4_ production pathway in the deep aquifers tested. However, the potential for CH_4_ production by H_2_-utilizing methanogens was not confirmed in the cultures using groundwater samples amended with H_2_/CO_2_. This may have been due to the growth inhibition of H_2_-utilizing methanogens caused by a shortage of inorganic nutrients, such as phosphate, vitamin, and trace elements, or by a high concentration of H_2_ and CO_2_ (41).

Although archaeal 16S rRNA genes closely related to the order *Methanobacteriales* were obtained from all sites (Fig. 4A), FISH-positive *Methanobacteriales* cells were only detected from ARM (Table 2). This result may have been due to the low abundance of *Methanobacteriales* cells in groundwater samples or a mismatch between the probe used in the present study and their 16S rRNA sequences.

The NGS analysis targeting bacterial 16S rRNA genes revealed the presence of bacteria belonging to *Firmicutes* in all groundwater samples (Fig. 4B). These bacteria are generally known to have the ability to degrade organic matter to H_2_ and CO_2_ by fermentation (21, 28, 30). Additionally, the members of the bacterial groups belonging to *Betaproteobacteria*, *Gammaproteobacteria*, *Actinobacteria*, *Chloroflexi*, and *Bacteroidetes*, which were also identified in each site, have been shown to possess the ability to grow by fermentation under anaerobic environments (5, 6, 20, 37, 54). We confirmed a high potential for H_2_ and CO_2_ production by H_2_-producing fermentative bacteria in the cultures using groundwater samples amended with YPG medium and BES (Fig. 5A). Therefore, these bacteria are considered to grow by fermentation and degrade organic matter to H_2_ and CO_2_ in the deep aquifers.

The H_2_-utilizing methanogenic archaea and H_2_-producing fermentative bacteria identified in the present study have frequently been found in subsurface oil reservoirs, natural gas reservoirs, and coal deposits in which microbial CH_4_ production has been observed (32, 33). Additionally, it is generally known that a syntrophic consortium of H_2_-producing fermentative bacteria and H_2_-utilizing methanogenic archaea leads to the biodegradation of organic matter to CH_4_ in anaerobic environments (38, 41). In the present study, a high potential for CH_4_ production by a syntrophic consortium of H_2_-producing fermentative bacteria and H_2_-utilizing methanogenic archaea was demonstrated by the cultures using groundwater samples amended with YPG medium (Fig. 5B). The potential for microbial CH_4_ production was similar to that previously reported in deep aquifers of the Paleogene accretionary prism (27, 32). The predominance of H_2_-utilizing methanogenic archaea and H_2_-producing fermentative bacteria in the cultures was also confirmed by the 16S rRNA gene analysis (Table S6). Although the predominant fermentative bacteria in the cultures belonged to the orders *Bacillales*, *Synergistales*, *Clostridiales*, and *Thermotogales*, except for *Clostridiales*, these bacterial groups were rarely found in natural groundwater samples (Fig. 4B). This may have been due to the strong selective pressure caused by using very high concentrations of organic substrates.

Our geochemical and microbiological data strongly suggest the presence of a CH_4_ production process by a syntrophic consortium of H_2_-producing fermentative bacteria and H_2_-utilizing methanogenic archaea in deep aquifers of the Cretaceous accretionary prism in Okinawa Island, Japan. The microbial activity and CH_4_ production process revealed in this study were similar to those previously reported in deep aquifers of the Paleogene accretionary prism (27, 32). Since accretionary prisms are derived from ancient marine sediments scraped from the subducting ocean plate, the sediments contain layers of mudstone rich in complex organic matter (8, 22). This organic matter is considered to support the activity of a microbial community that generates CH_4_ in deep aquifers. Taken together, our results suggest that the ongoing biodegradation of organic matter makes a major contribution to CH_4_ production in deep aquifers of the Cretaceous accretionary prism as well as those of the Paleogene accretionary prism.

## Supplementary Material



## Figures and Tables

**Table 1:** Physicochemical parameters of groundwater and components of natural gas.

Site code	Groundwater				Natural gas				
	
	Temp. (°C)	pH	ORP (mV)	EC (mS m^−1^)	N_2_ (vol.%)	CH_4_ (vol.%)	C_2_H_6_ (vol.%)	C_3_H_8_ (vol.%)	C_1_/(C_2_ + C_3_)
YHN	53.7	7.1	−275	4,500	0.24	99.7	0.044	0.003	2,117
LSH	40.7	7.4	−179	3,200	5.94	94.0	0.012	<0.001	7,902
XTU	49.8	7.5	−223	3,090	6.20	93.8	0.019	<0.001	5,031
ARM	41.5	7.8	−250	878	6.25	93.7	0.013	<0.001	7,037

Abbreviations: ORP, oxidation-reduction potential; EC, electrical conductivity; C_1_, CH_4_; C_2_, C_2_H_6_; C_3_, C_3_H_8_.

**Table 2:** Microbial cell density and relative abundance of FISH-positive cells in groundwater.

Site code	Microbial cell density (cells mL^−1^)	FISH-positive cells^a^					

		*Archaea* (%)	*Bacteria* (%)	*Bacteria/Archaea*	*Methanobacteriales* (%)	*Methanomicrobiales* (%)	*Methanosarcinales* (%)
YHN	3.4×10^3^	n.d.	n.d.	no data	n.d.	n.d.	n.d.
LSH	1.2×10^5^	3.7	4.8	1.3	n.d.	n.d.	n.d.
XTU	1.2×10^5^	17.4	21.6	1.2	n.d.	n.d.	n.d.
ARM	4.9×10^4^	13.4	33.1	2.5	5.9	n.d.	n.d.

Abbreviation: n.d., not detected.

a The relative abundance of FISH-positive cells was assessed by the count ratio of FISH-positive cells to SYBR Green I-stained cells.
