# Supplementary material for: Geochemical and Microbiological Evidence for Microbial Methane Production in Deep Aquifers of the Cretaceous Accretionary Prism
Source: Microbes Environ. 2018 Jun 13;33(2):205–13. doi: 10.1264/jsme2.ME17199 (PMC6031385; doi:10.1264/jsme2.ME17199)
Supplement: Supplementary file 1 [file 33_205_s1.pdf]

Table S1. Location, structure, and deep aquifers of wells for sampling.

Site name	Site code	Latitude	Longitude	Well depth (Strainer depth) (m)	Formation of deep aquifer	Water pumping method
Yuinchi Hotel Nanjo	YHN	26°09'53.6"N	127°46'05.1"E	2,119 (1,510-2,119)	Nago Group and Shimajiri Group	WP
Loisir Hotel	LSH	26°12'50.6"N	127°39'54.3"E	800 (no data)	Nago Group	NWP
Xystus Urasoe	XTU	26°14'04.7"N	127°42'30.5"E	1,560 (1,274-1,527)	Nago Group	NWP
Natural hot spring Aroma	ARM	26°17'19.4"N	127°44'45.8"E	1,300 (1,054-1,252)	Nago Group	NWP

Abbreviation: WP, water pump; NWP, natural water pressure.

Table S2. The chemical characteristics of groundwater, normal seawater, and ancient seawater.

Site code	Na^{+} (mg L^{-1})	Ca^{2+} (mg L^{-1})	Mg^{2+} (mg L^{-1})	K^{+} (mg L^{-1})	NH_4^{+} (mg L^{-1})	Cl^{-} (mg L^{-1})	Br^{-} (mg L^{-1})	I^{-} (mg L^{-1})	F^{-} (mg L^{-1})	PO_4^{3-} (mg L^{-1})	NO_3^{-} (mg L^{-1})	SO_4^{2-} (mg L^{-1})	S^{2-} (mg L^{-1})	HCO_3^{-} (mg L^{-1})	Acetate (mg L^{-1})	Formate (mg L^{-1})	DOC (mg L^{-1})
YHN	10,000	540	170	59	46	16,000	96	86	<0.5	<0.5	<1.0	9.3	<0.5	200	<5.0	<5.0	17
LSH	7,000	460	54	78	18	11,000	58	33	1.8	<0.5	<1.0	<0.1	<0.5	120	<5.0	<5.0	0.5
XTU	6,400	480	64	66	18	10,000	56	32	2.1	<0.5	<1.0	<0.1	<0.5	100	<5.0	<5.0	0.4
ARM	1,800	32	9.6	17	6.4	2,400	13	9.0	2.8	<0.5	<1.0	0.1	<0.5	450	<5.0	<5.0	<0.3
Normal seawater[a]	10,781	412	1,283	399	NA	19,352	67	0.05	NA	NA	NA	2,712	NA	105	NA	NA	NA
Ancient seawater[b]	8,200	120	370	250	510	18,000	140	119	NA	2.7	<0.005	<0.5	NA	1,100	<0.1	NA	NA

Abbreviation: DOC, dissolved organic carbon; NA, not available.

[a]Taken from Millero *et al* . (2008) Deep Sea Res., Part I 55:50－72.

[b]Taken from Katayama *et al. (* 2015) ISME J. 9:436－446.

Table S3. Stable isotopic signatures of groundwater and natural gas.

Site code	Groudwater			Natural gas	
	δD of H_2O (‰, VSMOW)	$\delta^{18}O$ of H_2O (‰, VSMOW)	$\delta^{13}C_{DIC}$ (‰, VPDB)	$\delta^{13}C_{CH4}$ (‰, VPDB)	α_c[a]
YHN	−5.8	−1.6	−8.64	−57.2	1.052
LSH	−12.6	0.6	0.14	−40.1	1.042
XTU	−13.0	0.5	3.13	−40.9	1.046
ARM	−24.3	−3.3	3.70	−36.6	1.042

Abbreviation: DIC, dissolved inorganic carbon; VSMOW, Vienna Standard Mean Ocean Water; VPDB, Vienna Pee Dee Belemnite.

[a] $\alpha_c = (\delta^{13}C_{DIC} + 10^3)/(\delta^{13}C_{CH4} + 10^3)$.

Table S4. Number of prokaryotic 16S rRNA gene sequences derived from groundwater and statistical estimators.

Site code	Total of seaquences	No. of OTUs	Coverage	Chao 1	Shannon index
YHN	41,573	504	99.5%	742	4.57
LSH	51,920	332	99.8%	571	2.97
XTU	14,005	429	99.4%	497	5.62
ARM	23,073	196	99.8%	231	3.95

Abbreviations: OTU, operational taxonomic unit.

Table S5. Archaeal and bacterial 16S rRNA gene sequence derived from the cultures using YPG medium-amended groundwater.

Site code	OTU	Accession number	No. of clones	Phylogenetic group	Closest cultivated species (% identity)	Predicted metabolism
YHN	*Archaea*					
	YHN_cA01	LC179580	56	*Methanobacteriales*	*Methanothermobacter defluvii* (99)	HM
	Total		56			
	Bacteria					
	YHN_cB01	LC179581	43	*Bacillales*	*Paenibacillus thailandensis* (82)	FE
	YHN_cB02	LC179582	7	*Bacillales*	*Paenibacillus thailandensis* (80)	FE
	YHN_cB03	LC179583	3	*Clostridiales*	*Pelotomaculum thermopropionicum* (90)	FE
	YHN_cB04	LC179584	2	*Clostridiales*	*Desulfotomaculum thermosapovorans* (94)	FE or SR
	Total		55			
LSH	*Archaea*					
	LSH_cA01	LC179571	53	*Methanobacteriales*	*Methanothermobacter defluvii* (99)	HM
	LSH_cA02	LC179572	2	*Methanobacteriales*	*Methanobacterium beijingense* (96)	HM
	Total		55			
	Bacteria					
	LSH_cB01	LC179573	31	*Synergistales*	*Thermovirga lienii* (92)	FE
	LSH_cB02	LC179574	17	*Limnochordales*	*Limnochorda pilosa* (99)	FE
	LSH_cB03	LC179575	6	*Clostridiales*	*Tissierella creatinophila* (84)	FE
	Total		54			
XTU	*Archaea*					
	XTU_cA01	LC179576	48	*Methanobacteriales*	*Methanothermobacter defluvii* (99)	HM
	Total		48			
	Bacteria					
	XTU_cB01	LC179577	29	*Clostridiales*	*Christensenella minuta* (86)	FE
	XTU_cB02	LC179578	23	*Ignavibacteriales*	*Melioribacter roseus* (99)	FE
	XTU_cB03	LC179579	4	*Clostridiales*	*Desulfonosporus thiosulfogenes* (91)	FE
	Total		56			
ARM	*Archaea*					
	ARM_cA01	LC179566	48	*Methanobacteriales*	*Methanothermobacter thermautotrophicus* (99)	HM
	Total		48			
	Bacteria					
	ARM_cB01	LC179567	39	*Thermotogales*	*Thermotoga elfii* (94)	FE
	ARM_cB02	LC179568	7	*Ignavibacteriales*	*Ignavibacterium album* (97)	FE
	ARM_cB03	LC179569	4	*Clostridiales*	*Tissierella creatinophila* (84)	FE
	ARM_cB04	LC179570	1	*Rhodocyclales*	*Thauera linaloolentis* (95)	DE
	Total		51			

Abbreviation: OTU, operational taxnomic unit; HM, H_2-utilizing methanogenesis; SR, sulfate-reduction; FE, fermentation; DE, denitrification.

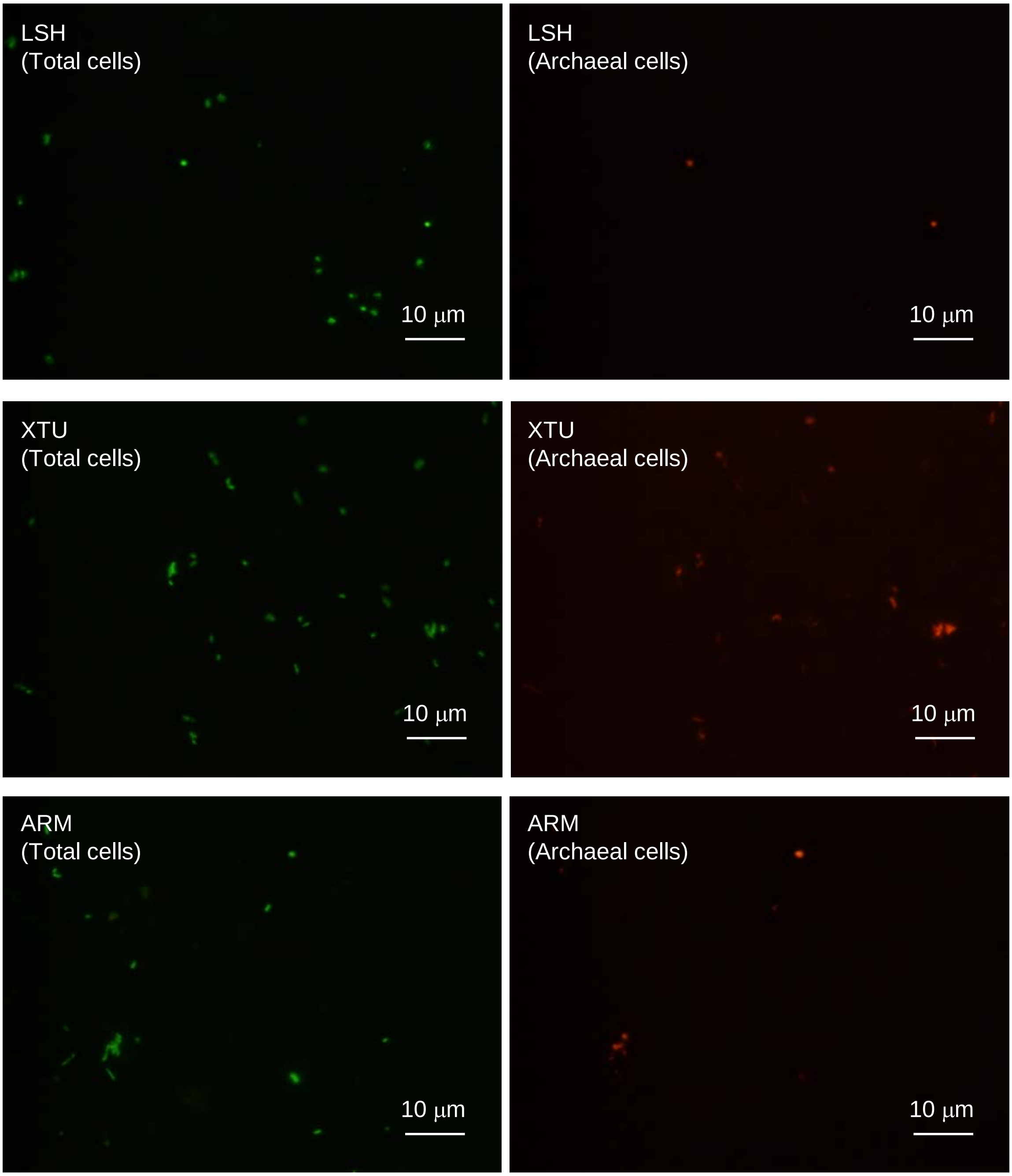

Fig. S1. Photomicrographs of FISH-positive archaeal cells in anaerobic groundwater samples. Each double panel shows identical fields and depicts total microbial cells counterstained with SYBR Green I (green) and FISH-positive cells stained with Cy3 (red) hybridized with ARCH915 (specific for the 16S rRNA of domain *Archaea*).

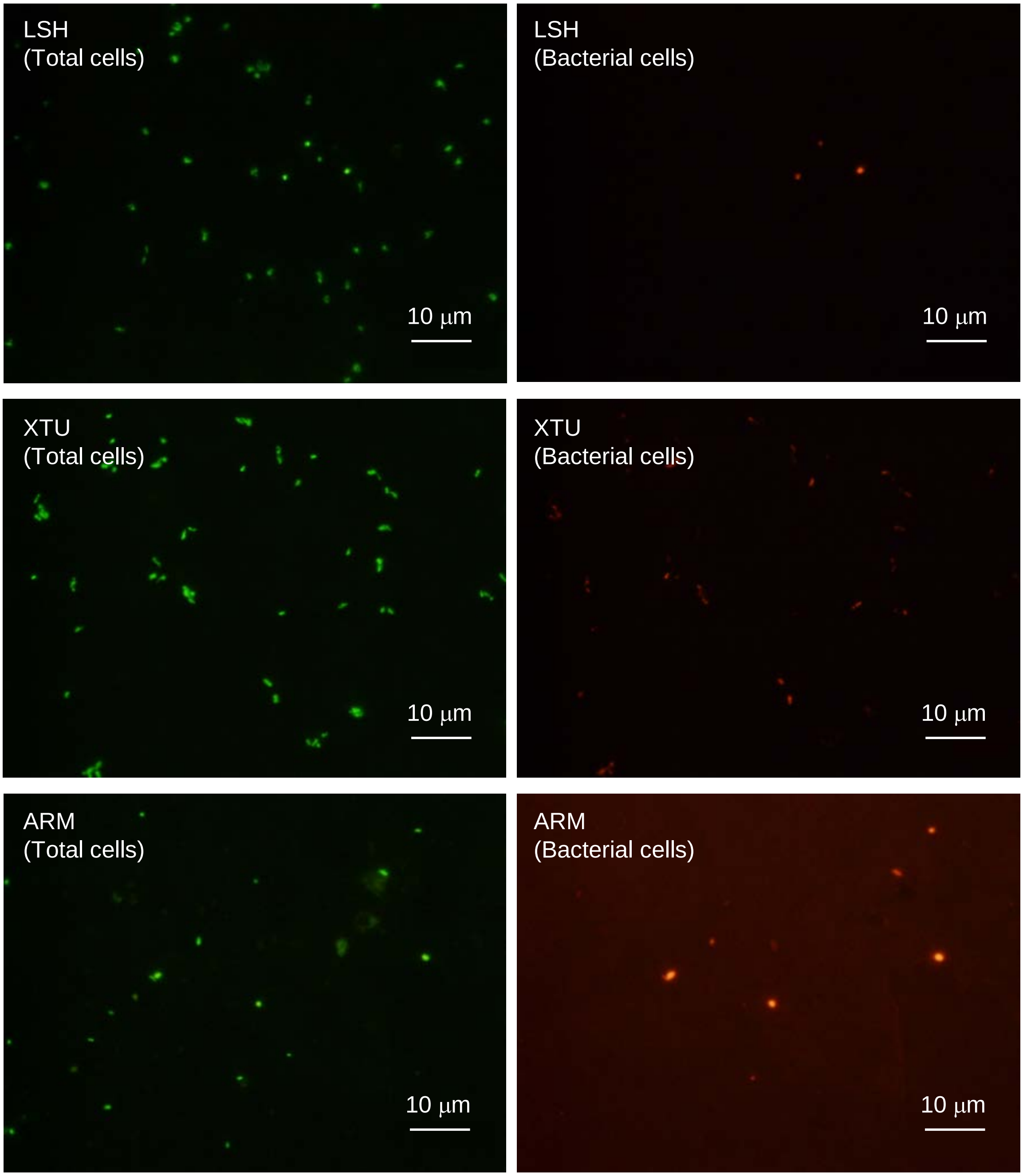

Fig. S2. Photomicrographs of FISH-positive bacterial cells in anaerobic groundwater samples. Each double panel shows identical fields and depicts total microbial cells counterstained with SYBR Green I (green) and FISH-positive cells stained with Cy3 (red) hybridized with EUB338 (specific for the 16S rRNA of domain *Bacteria*).

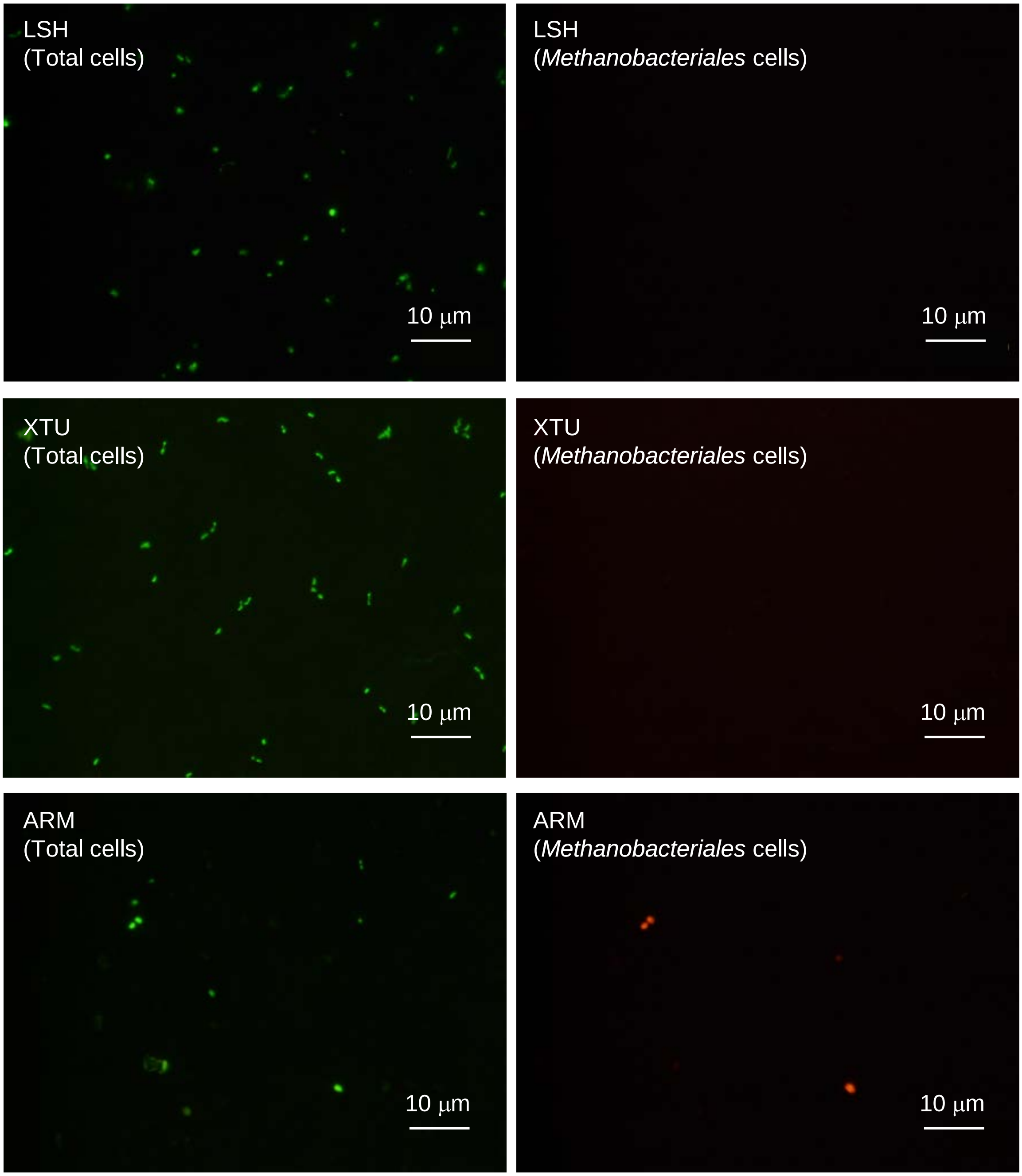

Fig. S3. Photomicrographs of FISH-positive *Methanobacteriales* cells in anaerobic groundwater samples. Each double panel shows identical fields and depicts total microbial cells counterstained with SYBR Green I (green) and FISH-positive cells stained with Cy3 (red) hybridized with MB1174 (specific for the 16S rRNA of the archaeal members of the order *Methanobacteriales*).

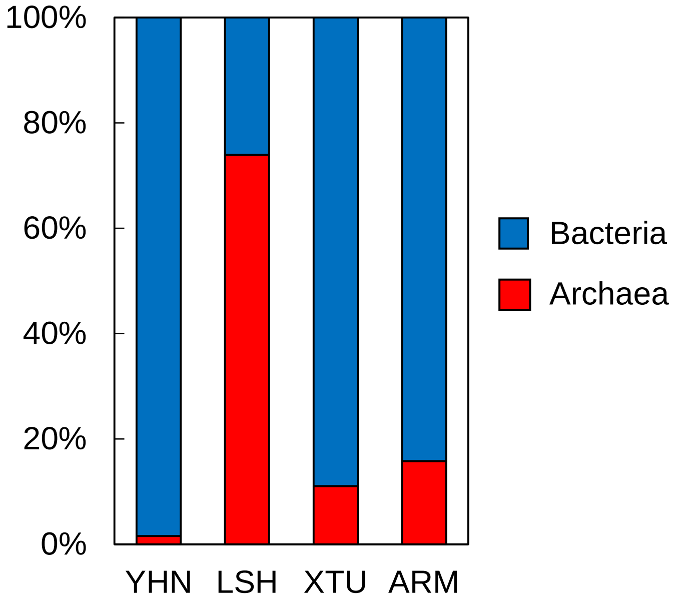

Fig. S4. Percent relative abundance of archaeal and bacterial 16S rRNA genes obtained from NGS analysis.

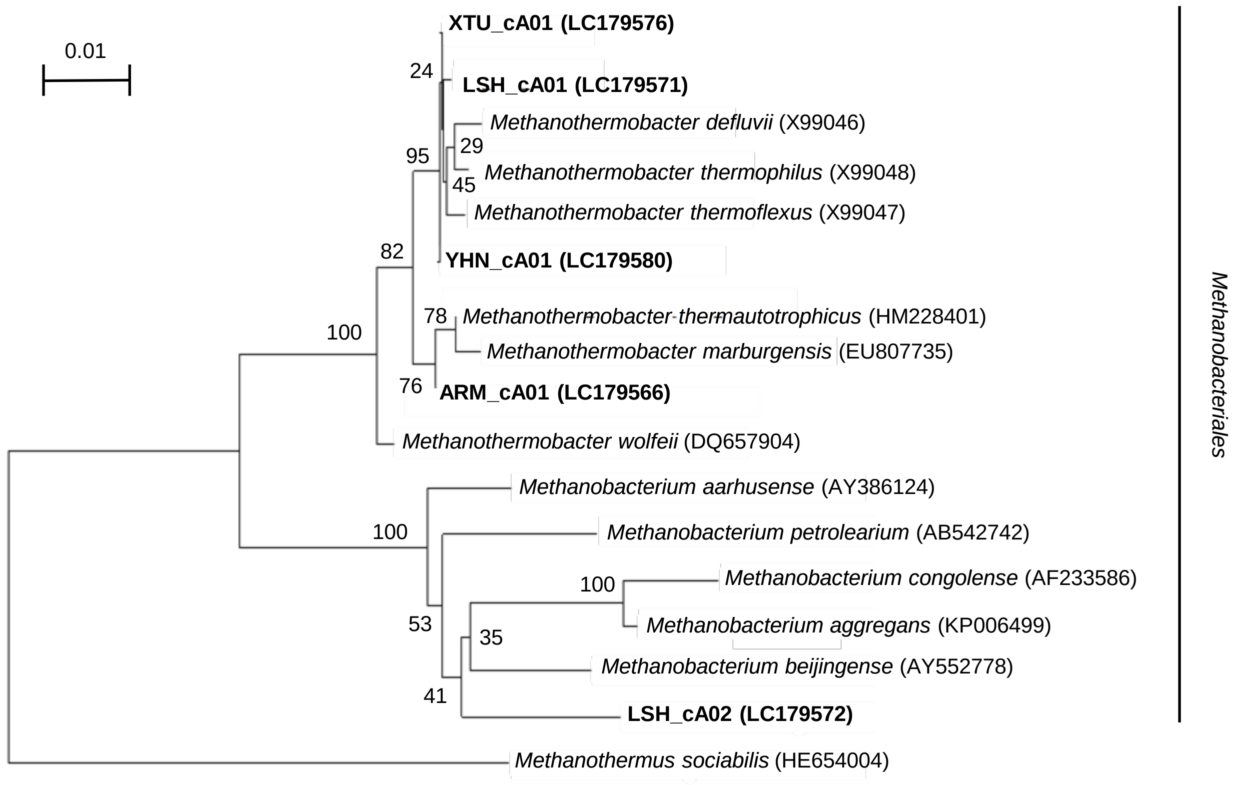

Fig. S5. Neighbor-joining tree of archaeal 16S rRNA gene sequences derived from the cultures using YPG medium-amended groundwater. The 5 OTUs obtained in this study are shown with their relatives. Accession numbers are shown in parentheses. Bootstrap values determined from 1,000 iterations are indicated at the branching points. The sequence of *Methanothermus sociabilis* was used as the outgroup to root the tree. The scale bar represents 1 substitution per 100 nucleotides.

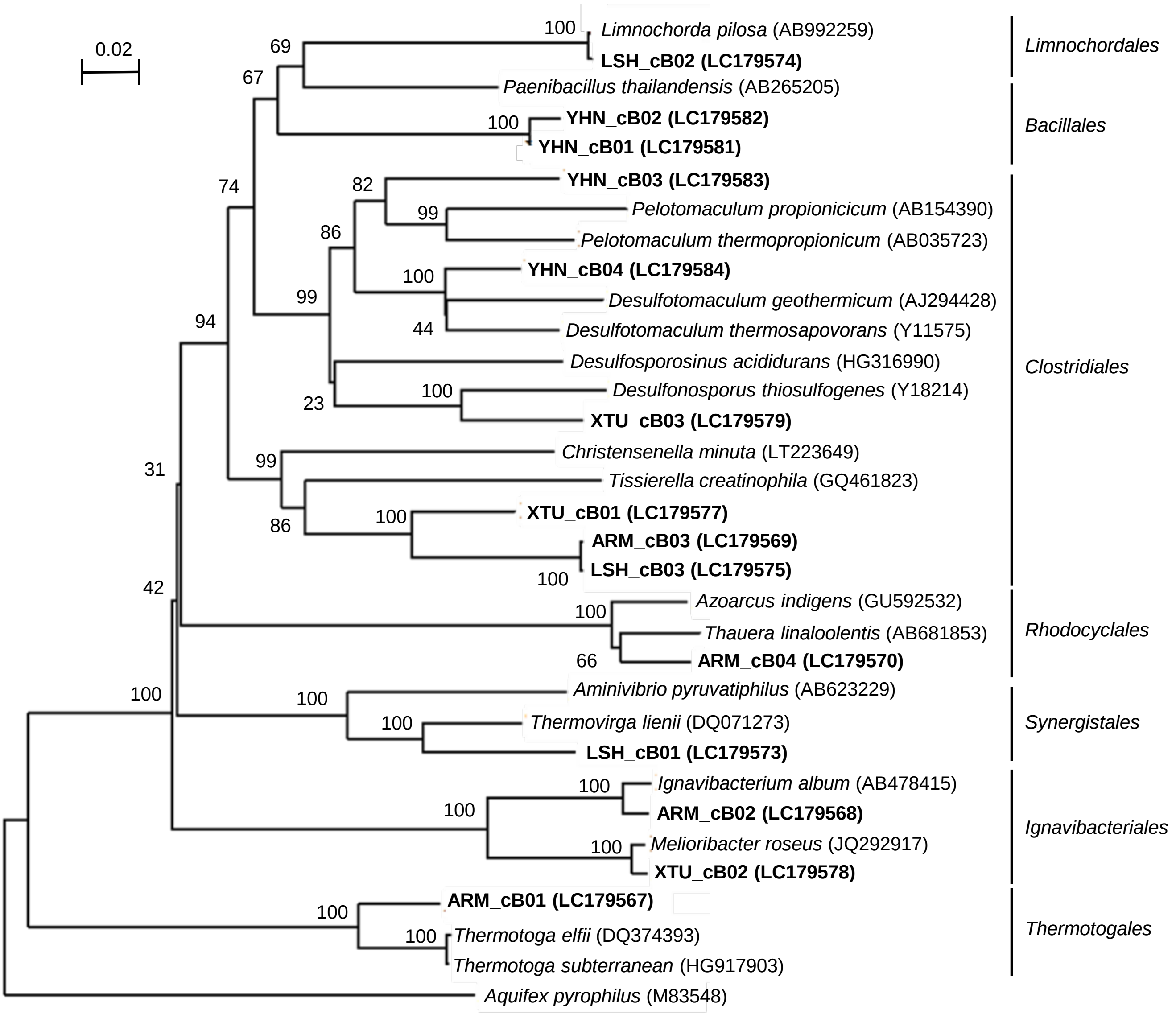

Fig. S6. Neighbor-joining tree of bacterial 16S rRNA gene sequences derived from the cultures using YPG medium-amended groundwater. The 14 OTUs obtained in this study are shown with their relatives. Accession numbers are shown in parentheses. Bootstrap values determined from 1,000 iterations are indicated at the branching points. The sequence of *Aquifex pyrophilus* was used as the outgroup to root the tree. The scale bar represents 2 substitutions per 100 nucleotides.
